# Trends in migraine and tension-type headaches in South Asia: findings from the Global Burden of Disease Study 2021 (1990–2021)

**DOI:** 10.3389/fneur.2025.1514712

**Published:** 2025-03-05

**Authors:** Prakasini Satapathy, Shubham Chauhan, Shilpa Gaidhane, Ashok Kumar Bishoyi, G. Padma Priya, Karthikeyan Jayabalan, Swati Mishra, Shilpa Sharma, Ganesh Bushi, Muhammed Shabil, Rukshar Syed, Kamal Kundra, Navneet Dev, Sabah Ansar, Sanjit Sah, Quazi Syed Zahiruddin, Shailesh Kumar Samal, Diptismita Jena, Khang Wen Goh

**Affiliations:** ^1^Centre of Research Impact and Outcome, Chitkara University, Rajpura, India; ^2^Faculty of Data Science and Information Technology, INTI International University, Nilai, Malaysia; ^3^Center for Global Health Research, Saveetha Medical College and Hospital, Saveetha Institute of Medical and Technical Sciences, Saveetha University, Chennai, India; ^4^One Health Centre (COHERD), Jawaharlal Nehru Medical College, Datta Meghe Institute of Higher Education, Wardha, India; ^5^Marwadi University Research Center, Department of Microbiology, Marwadi University, Rajkot, India; ^6^Department of Chemistry and Biochemistry, School of Sciences, JAIN (Deemed to be University), Bangalore, India; ^7^Department of Chemistry, Sathyabama Institute of Science and Technology, Chennai, India; ^8^Department of Pharmacology, IMS and SUM Hospital, Siksha 'O' Anusandhan (Deemed to be University), Bhubaneswar, India; ^9^Department of Pharmacy, Chandigarh Pharmacy College, Chandigarh Group of Colleges-Jhanjeri, Mohali, India; ^10^School of Pharmaceutical Sciences, Lovely Professional University, Phagwara, India; ^11^Research and Enterprise, University of Cyberjaya, Persiaran Bestari, Cyber, Cyberjaya, Malaysia; ^12^University Center for Research and Development, Chandigarh University, Mohali, India; ^13^Department of Medical Laboratories Techniques, AL-Mustaqbal University, Hillah, Iraq; ^14^IES Institute of Pharmacy, IES University, Bhopal, India; ^15^New Delhi Institute of Management, New Delhi, India; ^16^Department of Dermatology, Graphic Era Deemed to be University, Dehradun, India; ^17^Department of Clinical Laboratory Sciences, College of Applied Medical Sciences, King Saud University, Riyadh, Saudi Arabia; ^18^Department of Paediatrics, Dr. D. Y. Patil Medical College Hospital and Research Centre, Dr. D. Y. Patil Vidyapeeth (Deemed to be University), Pimpri-Chinchwad, India; ^19^Department of Public Health Dentistry, Dr. D. Y. Patil Medical College Hospital and Research Centre, Dr. D. Y. Patil Vidyapeeth (Deemed to be University), Pimpri-Chinchwad, India; ^20^Department of Medicine, Korea University, Seoul, Republic of Korea; ^21^South Asia Infant Feeding Research Network (SAIFRN), Global Consortium of Public Health and Research, Division of Evidence Synthesis, Datta Meghe Institute of Higher Education, Wardha, India; ^22^Unit of Immunology and Chronic Disease, Institute of Environmental Medicine, Karolinska Institute, Stockholm, Sweden; ^23^Noida Institute of Engineering and Technology (Pharmacy Institute), Greater Noida, India; ^24^Faculty of Data Science and Information Technology, INTI International University, Nilai, Malaysia; ^25^Faculty of Mathematics and Natural Sciences, Universitas Negeri Padang, Padang, Indonesia

**Keywords:** headache disorders, migraines, tension-type headaches, South Asia, years lived with disability, disease burden, global burden of disease

## Abstract

**Background:**

Headache disorders, including migraines and tension-type headaches (TTH), are major contributors to global disability. In South Asia, where these conditions are often underdiagnosed, their burden has grown substantially. This study evaluates trends in headache disorders across eight South Asian countries from 1990 to 2021, using data from the Global Burden of Disease (GBD) Study 2021.

**Methods:**

Data from the GBD study were analysed to evaluate incidence, prevalence, and years lived with disability (YLDs) for headache disorders and their subtypes. Trends were assessed using absolute numbers and age-standardized rates, with demographic patterns by age and gender examined to identify vulnerable populations. Joinpoint regression analysis was employed to detect significant temporal shifts.

**Results:**

From 1990 to 2021, headache disorders in South Asia rose from 114.2 million to 206.8 million in incidence, and from 367.4 million to 698.5 million in prevalence, with YLDs nearly doubling from 6.0 million to 11.6 million. Migraines accounted for 294.4 million cases, while TTH contributed 495.4 million cases, with YLDs increasing by 92.88 and 99.35%, respectively. Afghanistan saw the highest relative growth, while India contributed the largest absolute burden. Women and middle-aged adults were disproportionately affected, with the highest prevalence observed in females aged 30–34 years. The Maldives showed dramatic increases in TTH-related YLDs, highlighting disparities in smaller nations.

**Conclusion:**

The rising burden of headache disorders in South Asia highlights the need for region-specific strategies targeting high-burden subtypes, countries, and vulnerable populations to mitigate their disabling impacts.

## 1 Introduction

In 2021, headache disorders impacted approximately 40% of the global population, affecting around 3.1 billion people (1). Headache disorders rank among the most prevalent conditions affecting the nervous system. Migraine and TTH are among the most prevalent neurological disorders worldwide, significantly contributing to disability (1). These conditions constitute a significant public health burden due to their high prevalence and considerable socioeconomic impact (2). According to the International Headache Society (IHS), migraine is a primary headache disorder characterized by recurring episodes of severe pain, while TTH typically presents as a milder, bilateral headache without the associated symptoms of migraine (3). Although TTH generally causes less severe symptoms, it affects a larger portion of the population, while migraine is typically more disabling (4).

Migraine remains a leading cause of disability and lost productivity worldwide. Moreover, its attacks are complex neurological events that can last from hours to days, significantly impacting individuals’ ability to function in daily life (5, 6). Both genetic and environmental factors contribute to the onset of migraines, alongside stress, sleep disturbance, exhaustion, hormonal influences, dietary habits, and lifestyle triggers (7–9). Treatment options range from acute medications to preventive therapies, as well as lifestyle modifications aimed at improving quality of life. Despite the availability of these treatments, headache disorders remain underdiagnosed and undertreated, particularly in low- and middle-income countries (LMICs), where healthcare access is limited (10). They contribute significantly to socioeconomic burdens through lost productivity and increased healthcare costs (2, 11). Tension-type headaches, while less debilitating than migraines, are the most common form of primary headaches, more recent studies suggest that TTH affects approximately 26–38% of the global population (12). In South Asia, limited healthcare access and financial challenges, particularly among low-income populations, hinder the effective management of tension-type headaches. These constraints result in inadequate treatment and worsening health outcomes (13).

These disorders disproportionately affect individuals in lower socioeconomic conditions, where stressors such as food insecurity, unemployment, and limited healthcare access exacerbate their severity (14). Moreover, individuals with inadequate medical care are at higher risk of developing medication overuse, including opioids (15). Despite the recognized global burden of headache disorders, there remains a significant research gap in fully understanding these conditions in South Asia, especially using the most recent data. While previous studies have primarily focused on high-income countries, the region has remained underrepresented particularly in terms of long-term, comprehensive analysis utilizing data such as the GBD 2021. This study aims to fill a critical research gap by providing an in-depth epidemiological analysis of headache disorders and its subtypes in South Asia from 1990 to 2021. It examines trends in prevalence, incidence, and YLD, with a focus on identifying the most affected demographic groups. The findings offer valuable insights into the burden of these disorders, guiding the development of public health interventions tailored to the specific needs of various populations in the region.

## 2 Methods

### 2.1 Data sources

This study utilized data from the GBD Study 2021 to examine the burden of headache disorders, including migraines and TTH, in South Asia from 1990 to 2021. The GBD 2021 database consolidates information from diverse sources, such as national health surveys, hospital records, disease registries, and published epidemiological research, utilizing a standardized methodology to ensure consistency and comparability across regions and time periods. For this analysis, data were retrieved for eight South Asian countries, as classified by the World Bank: Afghanistan, Bangladesh, Bhutan, India, Maldives, Nepal, Pakistan, and Sri Lanka from the GBDx portal.^1^ The focus was on age-standardized rates and the absolute number of cases for incidence, prevalence, and YLDs for headache disorders and their subtypes, including migraines and TTH, offering a comprehensive view of their trends and burden across the region.

### 2.2 Statistical analysis

The Trends in the burden of headache disorders were analyzed using both absolute numbers and age-standardized rates to account for population growth and demographic differences. Joinpoint regression analysis was employed to detect significant temporal changes in age-standardized incidence (ASIR), prevalence (ASPR), and years lived with disability (ASYR). The joinpoint analysis was conducted via Joinpoint software (version 5.2.0) of the National Cancer Institute. Additionally, comparisons were made across the eight South Asian countries Afghanistan, Bangladesh, Bhutan, India, Maldives, Nepal, Pakistan, and Sri Lanka to assess variations in relative and absolute burdens. The analysis also distinguished trends for headache subtypes, migraines and TTH, to provide insights into their specific contributions to the overall burden. This approach facilitated the identification of regional disparities and country-specific patterns, offering a robust basis for understanding the dynamics of headache disorders in South Asia. QGIS (version 3.34.9) was used for spatial analysis and mapping to visualize geographic trends across South Asian countries, providing a clearer representation of the spatial distribution and variations in headache disorder burden over time. Boundary maps for the ASIRs, ASPRs, and ASYRs of headache disorders in South Asia were developed using QGIS software to reflect the different rates.

## 3 Results

### 3.1 Disease burden of headache disorders and subtypes in South Asia (1990–2021)

**Figure 1:**
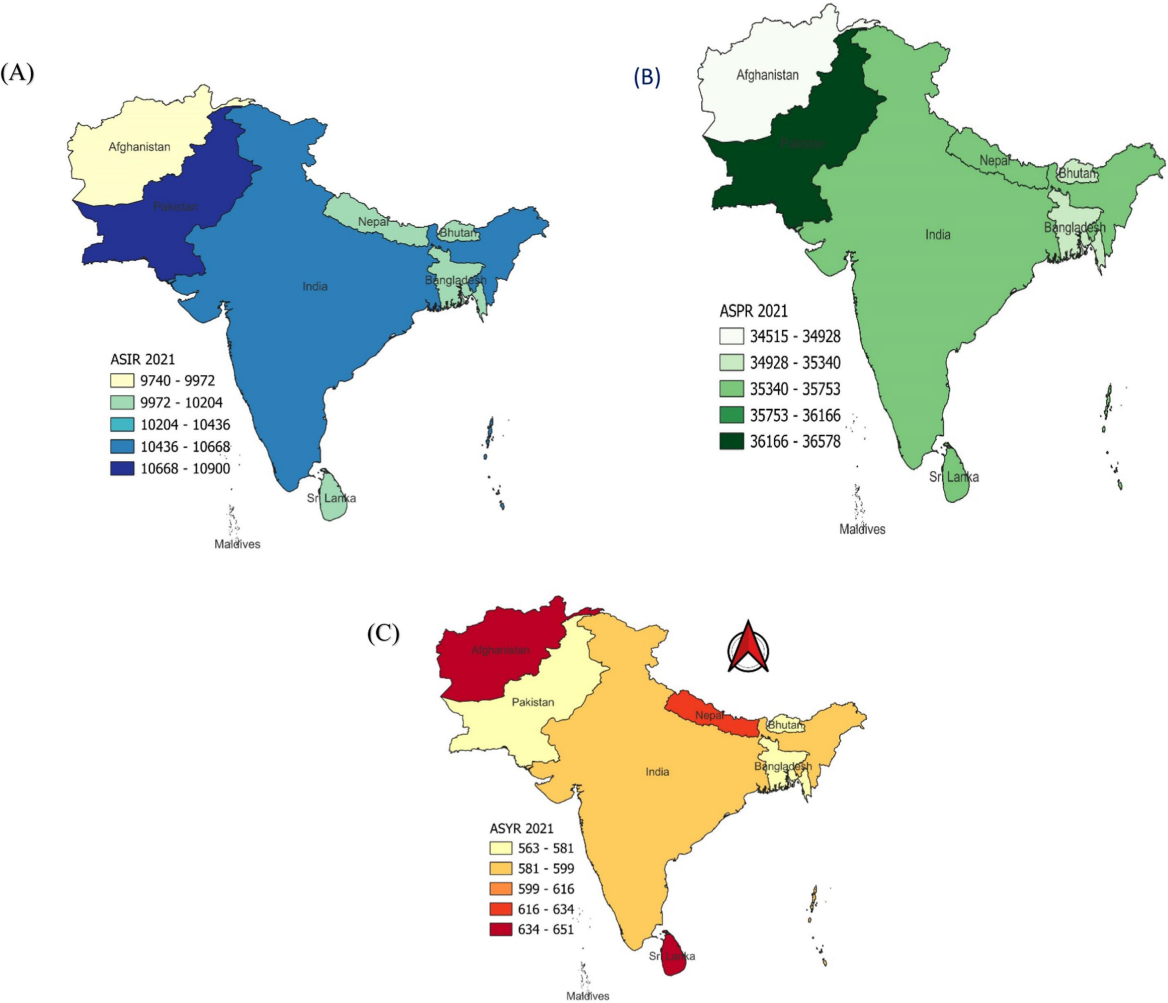
Spatial analysis of headache disorders in south Asia **(A)** The ASR of headache disorders incidence in 2021 **(B)** The ASR of headache disorders prevalence in 2021 **(C)** The ASR of headache disorders years lived with disability in 2021.

From 1990 to 2021, the burden of headache disorders, including migraines and TTH, increased significantly in South Asia. The region experienced an 81.03% increase in incidence, a 90.1% rise in prevalence, and a 93.41% growth in YLDs. Among countries, Afghanistan reported the highest relative increases, with incidence rising by 217.12%, prevalence by 211.32%, and YLDs by 215.4%. India prevalence increasing from 283,186.46 × 10^3^ to 527,359.45 × 10^3^, reflecting an 86.22% rise (Table 1). Bangladesh showed an 83.66% increase in YLDs, while the Maldives recorded a dramatic relative increase in headache-related YLDs, rising by 216.61%. At a regional level, incidence grew by 67.91%, prevalence by 91.85%, and YLDs by 92.88%. TTH, with incidence increasing by 82.98%, prevalence by 89.67%, and YLDs by 99.35%. The Maldives exhibited relative increase in TTH-related YLDs, rising by 242.47%. In contrast, age-standardized metrics showed stability over the study period. South Asia recorded minimal changes in age-standardized incidence rate (ASIR, 0.05%), prevalence rate (ASPR, 0.12%), and YLD rate (ASYR, 0.65%). Afghanistan and Pakistan showed negligible changes in ASIR, while Afghanistan exhibited a slight ASPR increase of 0.38%. Minor ASPR declines were observed in Bangladesh (−0.58%) and the Maldives (−0.27%). ASYR decreased in Afghanistan (−2.23%) but rose slightly in Bangladesh (1.62%). For migraines, South Asia’s age-standardized metrics showed minor fluctuations, with ASIR rising by 0.18%, ASPR by 0.38%, and ASYR by 0.66% (Figure 1 and Table 2). The Maldives recorded declines in ASIR (−3.34%) and ASPR (−4.14%). TTH metrics remained stable, with ASIR increasing by 0.03%, ASPR by 0.01%, and ASYR by 0.53%. The Maldives showed modest decreases in TTH ASIR (−0.77%) and ASPR (−0.83%).

**Table 1:** Incidence, prevalence, YLD, and annual percentage changes of headache disorders and subtypes in South Asia, 1990–2021.

	1990	2021	1990	2021	1990	2021	1990–2021	1990–2021	1990–2021
	Incidence numbers × 10^3 (95% UI)	Incidence numbers × 10^3 (95% UI)	Prevalence numbers × 10^3 (95% UI)	Prevalence numbers × 10^3 (95% UI)	YLD numbers × 10^3 (95% UI)	YLD numbers × 10^3 (95% UI)	% change in incidence numbers	% change in Prevalence numbers	% change in YLD numbers
Headache disorders									
Country/Region									
Afghanistan	886.64 (782.13–989.97)	2811.67 (2459.16–3183.87)	2980.55 (2698.68–3288.42)	9278.92 (8292.38–10301.64)	56.16 (12.17–121.32)	177.12 (36.81–381.93)	217.12 (204.89–228.61)	211.32 (202.25–220.2)	215.4 (192.4–230.44)
Male	408.51 (358.23–457.2)	1380.36 (1192.62–1568.94)	1298.87 (1158.67–1448.92)	4343.48 (3835.52–4885.88)	20.46 (4.61–43.43)	70.82 (14.73–151.92)	237.9 221.89–254.04)	234.4 (221.66–247.56)	246.21 (213.52–270.22)
Female	478.13 (422.63–532.75)	1431.31 (1254.04–1611.46)	1681.67 (1527.51–1850.72)	4935.44 (4460.56–5450.92)	35.7 (7.62–77.27)	106.3 (21.63–229.94)	199.36 (189.65–207.87)	193.48 (186.52–200.09)	197.75 (176.03–212.79)
Bangladesh	10387.73 (9111.78–11739.53)	17144.75 (15138.99–19137.48)	33236.25 (29797.54–36890.28)	59233.02 (53915.83–65157.72)	536.16 (72.06–1201.89)	984.73 (154.12–2160.48)	65.05 (58.4–71.75)	78.22 (72.81–83.21)	83.66 (74.15–112.98)
Male	5194.65 (4563.27–5830.41)	8244.51 (7284.82–9218.07)	16156.63 (14323.01–18114.81)	27349.64 (24800.74–30411.12)	226.6 (30.39–499.5)	390.97 (60.69–846.19)	58.71 (52.94–64.6)	69.28 (64.35–74.1)	72.54 (61.74–98.94)
Female	5193.07 (4568.01–5886.96)	8900.23 (7851.22–9988.18)	17079.62 (15259.79–18835.8)	31883.38 (28975.67–34978.78)	309.56 (41.84–695.41)	593.76 (94.32–1304.66)	71.39 (63.54–79.25)	86.67 (81.02–92.73)	91.81 (80.43–123.17)
Bhutan	62.16 (54.31–70.17)	80.03 (70.46–90.18)	200.89 (179.4–223.45)	279.14 (253.95–308.37)	3.25 (0.43–7.36)	4.66 (0.71–10.16)	28.74 (22.11–35.14)	38.95 (33.59–43.98)	43.17 (35.31–66.1)
Male	32.5 (28.24–36.67)	41.09 (36.14–46.45)	102.25 (90.11–115.35)	138.78 (125.15–154.85)	1.45 (0.19–3.25)	2.02 (0.3–4.4)	26.43 (19.43–33.06)	35.73 (29.88–41.28)	39.51 (29.8–62.55)
Female	29.67 (26–33.53)	38.95 (34.22–43.82)	98.64 (88.29–108.66)	140.35 (127.65–154.15)	1.8 (0.24–4.07)	2.63 (0.41–5.75)	31.28 (24.8–37.49)	42.29 (37.54–47.15)	46.1 (37.56–69.32)
India	87898.29 (77770.75–98212.71)	155679.8 (137968.91–173531.98)	283186.46 (259576.27–307867.72)	527359.45 (487064.98–569872.94)	4663.2 (732.79–10323.04)	8854.73 (1496.15–19077.44)	77.11 (72.86–81.35)	86.22 (82.56–90.06)	89.89 (81.48–107.26)
Male	44423.61 (39135.39–49955.91)	77906.16 (68622.79–87000.06)	137771.3 (126182.05–150321.69)	252588.45 (232106.29–274001.32)	1968.41 (323.6–4269.84)	3689.63 (617.77–7850.6)	75.37 (71.35–79.25)	83.34 (79.53–87.42)	87.44 (75.91–103.16)
Female	43474.68 (38564.88–48433.19)	77773.63 (69002.23–86530.64)	145415.16 (133658.22–157607.56)	274,771 (253524.58–296634.48)	2694.79 (417.01–6054.41)	5165.09 (883.26–11308.25)	78.89 (74.41–83.45)	88.96 (84.35–93.76)	91.67 (81.24–112.35)
Maldives	20.01 (17.43–22.66)	54.71 (47.63–62.25)	65.38 (58.75–72.55)	201.23 (181.36–222.82)	1.15 (0.14–2.53)	3.65 (0.55–7.79)	173.46 (149.55–199.15)	207.79 (189.87–226.4)	216.61 (192.6–276.29)
Male	9.56 (8.37–10.81)	32.62 (28.09–37.63)	30.43 (27.12–33.91)	118.79 (105.82–132.67)	0.47 (0.07–1.03)	2 (0.34–4.29)	241.28 (206–279.88)	290.38 (264.12–318.88)	320.7 (280.74–383.64)
Female	10.45 (9.19–11.82)	22.09 (19.4–24.89)	34.95 (31.32–38.51)	82.44 (75.25–90.2)	0.68 (0.08–1.51)	1.66 (0.22–3.62)	111.4 (96.69–125.85)	135.88 (124.38–147)	143.9 (126.37–187.77)
Nepal	1868.71 (1644.5–2118.55)	3225.48 (2848.6–3588.49)	6159.41 (5535.3–6824.32)	11224.29 (10182.7–12313.64)	104.26 (14.93–229.06)	195.5 (30.64–427.62)	72.6 (64.9–79.32)	82.23 (76.14–87.98)	87.51 (77.23–103.04)
Male	912.88 (796.39–1033.46)	1496.81 (1313.36–1677.76)	2901.86 (2577.04–3256.04)	4970.57 (4476.48–5485.36)	42.89 (6.07–95.72)	74.73 (11.25–167.42)	63.97 (56.07–71.48)	71.29 (63.46–78.83)	74.25 (62.03–94.38)
Female	955.83 (838.41–1081.69)	1728.67 (1535.85–1921.72)	3257.56 (2944.33–3593.87)	6253.72 (5672.79–6849.48)	61.38 (9–133.75)	120.77 (19.42–263.19)	80.86 (71.65–89.25)	91.98 (84.82–99.04)	96.78 (82.43–118.57)
Pakistan	11379.51 (10030.92–12670.62)	25522.1 (22488.81–28388.88)	35575.2 (32478.86–38724.81)	82578.25 (75438.28–89700.89)	549.11 (77.97–1235.6)	1277.31 (156.58–2848.57)	124.28 (119.43–129.26)	132.12 (129.18–135.3)	132.61 (103.42–139.71)
Male	5923.18 (5174.12–6647.32)	12943.26 (11301.43–14457.35)	18060.75 (16402.09–19794.96)	40579.61 (36893.47–44544.93)	242.97 (33.09–534.52)	544.15 (63.3–1208.19)	118.52 (114.19–123.1)	124.68 (121.92–127.61)	123.96 (91.21–132)
Female	5456.33 (4813.57–6065.6)	12578.84 (11185.42–14001.11)	17514.45 (16032.77–19023.46)	41998.64 (38532.45–45430.03)	306.14 (43.91–682.62)	733.15 (93.87–1617.84)	130.54 (124.84–136.25)	139.79 (136.4–143.2)	139.48 (114.59–148.24)
Sri Lanka	1732.19 (1527.27–1945.51)	2287.08 (2034.76–2542.82)	6043.7 (5509.57–6644.36)	8363.53 (7654.26–9168.61)	109.25 (14.74–237.19)	150.4 (23.25–320.27)	32.03 (27.55–36.9)	38.38 (34.44–42.13)	37.67 (31.29–55.39)
Male	810.37 (710.55–916.46)	1023.13 (902.48–1141.39)	2741.46 (2466.35–3048.16)	3606.18 (3263.51–3953.78)	43.7 (6.84–92.39)	57.44 (10.03–122.62)	26.25 (22.38–30.71)	31.54 (27.8–35.4)	31.43 (23.95–45.9)
Female	921.82 (816.22–1034.16)	1263.95 (1130.48–1399.46)	3302.24 (3000.88–3610.18)	4757.35 (4360.93–5195.1)	65.54 (8.03–145.09)	92.96 (13.42–202.23)	37.11 (32.01–42.69)	44.06 (40.04–48.23)	41.82 (35–62.91)
South Asia - WB	114235.24 (100894.46–127113.39)	206805.62 (183259.88–229763.48)	367447.84 (336843.49–400239.07)	698517.82 (644502.85–756514.1)	6022.54 (925.6–13357.81)	11648.08 (1890.93–25321.49)	81.03 (76.89–85.29)	90.1 (86.71–93.72)	93.41 (86.03–108.58)
Male	57715.26 (50913.33–64786.4)	103067.95 (90466.47–114777.54)	179063.55 (163587.69–196139.53)	333695.5 (305828.4–363694.9)	2546.95 (405.5–5528.95)	4831.76 (776.24–10408.69)	78.58 (74.56–82.38)	86.36 (82.82–89.9)	89.71 (80.06–102.31)
Female	56519.97 (49945.28–62725.18)	103737.67 (92095.06–115057.39)	188384.29 (172547.92–203895.47)	364822.32 (336003.24–393648.03)	3475.59 (530.51–7808.96)	6816.32 (1122.55–14912.8)	83.54 (79.15–88.14)	93.66 (89.44–97.91)	96.12 (87.42–112.98)
Migraine									
Afghanistan	126.54 (105.22–149.78)	423.39 (352.32–502.28)	1323.9 (1105.65–1572.23)	4189.02 (3477.68–5015.57)	50.87 (8.42–115.6)	161.05 (24.44–362.08)	234.59 (223.69–247.62)	216.41 (205.96–226.02)	216.6 (190.42–232.12)
Male	47.39 (38.2–57.48)	167.84 (135.92–201.93)	471 (386.48–561.64)	1629.85 (1325.5–1971.8)	18.29 (3.06–41.25)	63.67 (9.93–144.53)	254.17 (241.3–271.54)	246.04 (230.95–260.18)	248.11 (211.87–272.35)
Female	79.15 (66.54–92.54)	255.55 (214.59–300.03)	852.9 (714.81–1009.13)	2559.17 (2141.46–3018.41)	32.58 (5.3–74.54)	97.38 (14.41–220.55)	222.86 (212.41–235.14)	200.05 (191.31–208.03)	198.9 (173.6–214.42)
Bangladesh	1419.25 (1186.79–1668.5)	2106.74 (1788.77–2421.88)	13735.31 (11473.05–16531.21)	24942.55 (21005.79–29258.18)	494.38 (50.92–1130.53)	903.35 (108.41–2037.19)	48.44 (41–56.77)	81.59 (74.36–89.56)	82.72 (73.17–113.26)
Male	593.45 (494.74–700.21)	843.56 (714.96–978.72)	5725.64 (4745.86–6850.98)	9750.72 (8206.82–11513.69)	207.1 (21.49–485.95)	355.45 (41.46–820.46)	42.14 (35.57–49.31)	70.3 (64.55–76.75)	71.63 (61.44–99.15)
Female	825.8 (691.18–972.23)	1263.18 (1073.79–1461.59)	8009.67 (6618.89–9628.43)	15191.83 (12778.78–17946.64)	287.28 (29.11–650.75)	547.9 (66.8–1212.87)	52.96 (45.09–62.63)	89.67 (81.21–98.89)	90.72 (78.66–123.66)
Bhutan	8.39 (7.04–9.82)	9.64 (8.17–11.18)	82.94 (68.85–99.67)	117.27 (98.76–138.49)	3 (0.3–6.98)	4.27 (0.5–9.73)	14.8 (7.95–23.5)	41.38 (35.01–49.12)	42.46 (34.3–66.52)
Male	3.72 (3.11–4.39)	4.15 (3.51–4.83)	36.55 (30.07–43.98)	50.27 (42.39–59.5)	1.33 (0.13–3.15)	1.84 (0.21–4.26)	11.43 (4.64–20.13)	37.54 (30.53–45.55)	38.79 (28.8–63.44)
Female	4.67 (3.92–5.47)	5.49 (4.63–6.38)	46.39 (38.33–55.79)	66.99 (56.35–78.96)	1.67 (0.17–3.88)	2.43 (0.29–5.49)	17.48 (10.49–26.09)	44.41 (37.82–52.06)	45.38 (36.35–71.49)
India	11265.22 (9867.46–12808.61)	18411.21 (16138.21–20773.3)	118839.68 (101287.74–137816.95)	223124.2 (189835.22–254668.07)	4278.55 (497.81–9617.57)	8096.33 (1015.75–18217.29)	63.43 (56.44–70.49)	87.75 (78.96–95.6)	89.23 (80.64–107.76)
Male	4689.62 (4093.11–5344.41)	7674.41 (6678.93–8734.79)	49081.2 (41694.73–57182.91)	90998.72 (77378.27–104318.09)	1787.75 (219.61–4048.6)	3342.97 (424.38–7464.83)	63.65 (55.58–72.47)	85.4 (74.08–97.09)	86.99 (75–102.82)
Female	6575.6 (5772.11–7469.35)	10736.8 (9418.63–12031.02)	69758.48 (59540.03–80752.35)	132125.49 (113626.28–151556.1)	2490.8 (281.13–5651.14)	4753.36 (590.94–10718.97)	63.28 (55.53–72.43)	89.4 (78.6–99.42)	90.84 (80.15–112.7)
Maldives	3 (2.52–3.56)	6.78 (5.69–8.05)	28.85 (24.18–34.65)	89.87 (75.95–106.94)	1.07 (0.1–2.48)	3.37 (0.36–7.62)	125.91 (101.86–157.38)	211.45 (189.18–238.16)	214.63 (190.23–281.54)
Male	1.21 (1–1.45)	3.54 (2.92–4.3)	11.61 (9.64–13.97)	48.22 (40.64–57.95)	0.44 (0.05–0.98)	1.83 (0.21–4.06)	193.24 (153.26–246.35)	315.37 (278.73–354.42)	319.68 (278.93–383.83)
Female	1.8 (1.51–2.12)	3.25 (2.77–3.79)	17.25 (14.3–20.75)	41.65 (35.01–49.21)	0.64 (0.05–1.5)	1.54 (0.15–3.55)	80.71 (66.15–99.27)	141.49 (127.8–157.75)	142.77 (125.44–188.59)
Nepal	262.33 (219.54–306.73)	418.83 (356.19–488.55)	2682.91 (2238.42–3199.66)	4977.23 (4192.67–5913.1)	96.23 (10.29–217.72)	180.03 (20.9–408.34)	59.66 (50.68–68.49)	85.52 (76.44–94.15)	87.08 (76.13–105.95)
Male	108.33 (90.42–127.9)	164.67 (138.8–192.9)	1091.86 (892.94–1306.87)	1878.57 (1578.87–2234.12)	39.3 (4.12–91.69)	68.33 (7.62–156.85)	52.01 (43.42–61.69)	72.05 (61.88–82.53)	73.86 (61.01–95.01)
Female	154 (128.69–179.91)	254.16 (215.57–296.88)	1591.04 (1339.59–1896.6)	3098.66 (2613.37–3681.05)	56.93 (6.21–127.29)	111.7 (12.73–249.48)	65.04 (54.38–75.76)	94.76 (83.34–105.81)	96.2 (81.13–118.36)
Pakistan	1434.88 (1238.38–1637.27)	3125.8 (2729.57–3528.27)	14026.04 (11831.21–16136.2)	33211.06 (27967.36–38280.81)	504.2 (53.64–1155.36)	1178.39 (107.59–2697.29)	117.84 (112.25–123.91)	136.78 (132.93–140.84)	133.72 (101.91–140.22)
Male	629.64 (544.75–721.63)	1336.1 (1161.11–1515.37)	6134.5 (5159.89–7164.7)	13983.94 (11793.6–16314.93)	221.44 (22.76–503.32)	498.41 (42.32–1137.88)	112.2 (107.61–116.93)	127.96 (124.65–131.53)	125.07 (89.34–132.64)
Female	805.24 (694.23–920.66)	1789.69 (1559.93–2017.81)	7891.54 (6665.32–9053.99)	19227.13 (16318.65–22070.84)	282.75 (30.61–651.78)	679.98 (65.33–1557.9)	122.25 (116.11–129.39)	143.64 (139.23–148.35)	140.49 (112–148.67)
Sri Lanka	240.11 (203.71–279.98)	280.66 (240.57–323.34)	2715.64 (2281.88–3219.9)	3716.04 (3161.62–4382.23)	101.28 (10.37–232.56)	138.58 (16.23–312.07)	16.89 (13.27–20.89)	36.84 (31.58–42.34)	36.83 (30.44–57.87)
Male	95.73 (80.09–112.32)	109.08 (92.7–126.24)	1067.69 (896.89–1281.85)	1393.97 (1183.94–1645.56)	40.03 (4.75–88.94)	52.3 (7.11–115.5)	13.95 (10.56–17.63)	30.56 (25.74–35.66)	30.66 (23.41–47.59)
Female	144.38 (122.54–167.77)	171.58 (147.83–197.95)	1647.95 (1385.12–1961.5)	2322.07 (1973.36–2740.86)	61.25 (5.59–143.25)	86.28 (9.16–199.61)	18.84 (15.08–23.07)	40.91 (35.43–46.72)	40.87 (33.39–65.42)
South Asia	14759.73 (12887.91–16862.66)	24783.05 (21684.26–27909.5)	153435.28 (130208.04–178317.98)	294367.24 (250892.57–337527.98)	5529.57 (632.21–12439.58)	10665.37 (1293.68–23958.29)	67.91 (61.54–74.39)	91.85 (84.48–98.54)	92.88 (85.66–108.22)
Male	6169.08 (5372.68–7052.56)	10303.35 (8950.67–11732.91)	63620.05 (53811.54–73749.08)	119734.25 (101830.33–138223.52)	2315.68 (274.57–5276.75)	4384.8 (536.79–9751.05)	67.02 (60.08–74.36)	88.2 (78.92–97.37)	89.35 (79.45–101.61)
Female	8590.65 (7510.48–9814.44)	14479.7 (12697.15–16268.27)	89815.23 (76542.77–104491.5)	174632.99 (149512.62–199791.93)	3213.89 (360.14–7250.88)	6280.57 (756.8–14085.27)	68.55 (61.37–76.97)	94.44 (85.09–102.89)	95.42 (86.55–113.87)
TTH									
Afghanistan	760.1 (659.12–862.62)	2388.29 (2030.72–2745.79)	2017.13 (1723.47–2364.62)	6238.85 (5140.82–7393.95)	5.29 (1.66–16.35)	16.06 (4.93–50.94)	214.21 (199.99–227.88)	209.29 (192.18–224.23)	203.92 (161.12–233.61)
Male	361.12 (312.53–410.87)	1212.52 (1025.66–1,400)	953.45 (804.28–1121.89)	3155.16 (2611.65–3750.4)	2.16 (0.6–7.32)	7.15 (1.99–24.84)	235.77 (216.95–253.54)	230.92 (210.34–251.01)	230.17 (161.54–284.02)
Female	398.98 (343.19–453.57)	1175.76 (1002.24–1355.07)	1063.68 (907.1–1238.07)	3083.69 (2551.38–3662.7)	3.12 (1.02–9.04)	8.92 (2.91–25.53)	194.69 (183.01–205.18)	189.91 (176.61–201.68)	185.71 (144.5–222.05)
Bangladesh	8968.47 (7717.59–10244.93)	15038.01 (13060.55–17044.56)	23586.88 (19720.29–27923.63)	41769.7 (35462.42–48926.06)	41.78 (9.57–171.67)	81.38 (21.25–297.62)	67.68 (59.91–75.01)	77.09 (69.12–85.05)	94.81 (64.38–139.84)
Male	4601.2 (3978.02–5236.1)	7400.96 (6449.52–8376.83)	12153.97 (10081.71–14473.57)	20568.22 (17438.78–24217.95)	19.5 (4.16–94)	35.52 (8.26–150.97)	60.85 (54.46–67.38)	69.23 (62.31–76.39)	82.2 (56.28–134.56)
Female	4367.28 (3723.59–5039.66)	7637.05 (6616.86–8713.74)	11432.91 (9539.9–13552.34)	21201.48 (18000.03–24805.85)	22.28 (5.51–83.36)	45.86 (12.4–153.85)	74.87 (65.64–83.39)	85.44 (76.04–94.92)	105.84 (70.72–159.34)
Bhutan	53.77 (46.07–61.61)	70.4 (61.13–80.34)	142.73 (118.73–169.23)	197.37 (167.53–232.59)	0.25 (0.06–1.07)	0.38 (0.1–1.43)	30.92 (23.76–38.14)	38.29 (30.8–45.88)	51.55 (26.6–93.23)
Male	28.77 (24.6–32.9)	36.94 (32.02–42.41)	76.77 (63.19–91.88)	104.03 (87.6–122.93)	0.12 (0.03–0.63)	0.18 (0.04–0.78)	28.37 (20.52–35.74)	35.52 (27.3–43.82)	47.43 (24.05–94.49)
Female	25 (21.26–28.82)	33.46 (28.95–38.29)	65.96 (54.87–78.22)	93.34 (79.04–109.97)	0.13 (0.03–0.48)	0.2 (0.06–0.68)	33.86 (26.7–40.96)	41.51 (34.21–48.75)	55.43 (27.94–99.16)
India	76633.07 (66322.99–86338.29)	137268.58 (119809.09–154892.26)	200920.23 (175,296–229590.95)	373405.6 (329155.71–422556.13)	384.66 (99.77–1487.62)	758.4 (204.15–2689.27)	79.12 (74.41–83.51)	85.85 81.77–89.75)	97.16 (73.97–112.6)
Male	39733.99 (34342.29–44975.39)	70231.75 (61095.06–79587.39)	103763.44 (90308.8–118184.84)	189829.03 (166497.27–214918.98)	180.66 (44.43–813.39)	346.66 (85.51–1420.18)	76.75 (72.36–80.92)	82.94 (79.11–86.55)	91.89 (71.93–109.73)
Female	36899.08 (32083.5–41775.81)	67036.83 (58313.69–75577.78)	97156.79 (84140.78–112075.14)	183576.58 (160959.97–210047.91)	203.99 (56.8–708.36)	411.73 (117.98–1356.57)	81.68 (76.54–86.41)	88.95 (84.34–93.31)	101.84 (75.51–119.57)
Maldives	17.01 (14.38–19.59)	47.93 (41.07–55.4)	45 (37.37–52.93)	138.99 (116.42–162.99)	0.08 (0.02–0.34)	0.28 (0.08–1.05)	181.85 (153.41–211.19)	208.83 (182.75–239.76)	242.47 (186.82–337.68)
Male	8.35 (7.15–9.59)	29.09 (24.47–34.02)	22.03 (18.3–25.79)	84.85 (70.11–100.4)	0.04 (0.01–0.18)	0.17 (0.04–0.68)	248.22 (206.03–291.01)	285.21 (247.65–327.16)	331.97 (246.72–479.33)
Female	8.65 (7.31–9.97)	18.84 (16.23–21.54)	22.98 (19.02–27.31)	54.14 (46.09–62.61)	0.04 (0.01–0.16)	0.11 (0.03–0.4)	117.78 (100.05–135.31)	135.61 (118.73–153.75)	160.76 (111.31–241.02)
Nepal	1606.38 (1381.25–1839.81)	2806.65 (2439.48–3184.3)	4275.83 (3577.36–5019.81)	7729.04 (6506.17–9165.78)	8.03 (2.05–32.77)	15.47 (3.99–57.36)	74.72 (65.84–82.28)	80.76 (72.37–89.39)	92.71 (65.36–130.69)
Male	804.56 (692.49–926.19)	1332.14 (1150.69–1515.12)	2139.28 (1784.57–2553.25)	3662.65 (3083.07–4285.16)	3.59 (0.78–17.42)	6.4 (1.45–27.23)	65.57 (56.72–74.21)	71.21 (59.89–82.1)	78.52 (48.45–124.9)
Female	801.82 (682.68–922.14)	1474.51 (1285.38–1665.97)	2136.55 (1801.17–2498.91)	4066.39 (3428.53–4815.58)	4.44 (1.25–16.17)	9.07 (2.61–29.69)	83.89 (72.78–94.57)	90.33 (79.29–102.55)	104.17 (71.48–153.49)
Pakistan	9944.63 (8563.86–11207.09)	22396.3 (19364.05–25287.79)	26048.63 (22708.66–29825.2)	60143.15 (52453.13–68,879)	44.92 (10.49–191.51)	98.91 (21.95–418.39)	125.21 (119.45–130.76)	130.89 (126.63–135.89)	120.22 (83.18–145.55)
Male	5293.54 (4530.18–5987.77)	11607.15 (9920.23–13049.76)	13942.72 (12183.03–15927.99)	31250.07 (27306.86–35786.18)	21.53 (4.52–107.5)	45.74 (8.79–231.03)	119.27 (114.41–124.25)	124.13 (120.43–128.42)	112.48 (65.14–139.97)
Female	4651.09 (4009.97–5267.81)	10789.15 (9398.92–12227.92)	12105.91 (10526.39–13893.78)	28893.08 (24991.56–33136.47)	23.39 (5.88–86.15)	53.17 (13.15–205.02)	131.97 (125.23–138.59)	138.67 (133.31–144.36)	127.35 (83.56–154.57)
Sri Lanka	1492.08 (1280.04–1699.98)	2006.42 (1755.9–2256.62)	4148.91 (3504.4–4829.57)	5772.37 (4994.85–6593.05)	7.97 (2.07–30.4)	11.82 (3.2–42.49)	34.47 (29.28–40.6)	39.13 (33.21–45.86)	48.25 (26.03–75.21)
Male	714.64 (615.67–817.04)	914.05 (794.32–1033.52)	1976.21 (1656.3–2310.71)	2604.61 (2234.73–3000.74)	3.67 (0.9–15.5)	5.14 (1.3–19.14)	27.9 (23.55–33.23)	31.8 (26.35–37.86)	39.83 (19.3–74.82)
Female	777.43 (673.54–885.53)	1092.37 (959.98–1223.29)	2172.7 (1838.5–2524.63)	3167.76 (2718.35–3596.73)	4.3 (1.13–15.54)	6.68 (1.87–22.7)	40.51 (34.43–47.27)	45.8 (39.09–53.68)	55.45 (30.16–92.85)
South Asia	99475.51 (85883.35–111940.01)	182022.57 (158959.03–205016.74)	261185.34 (226165.84–300473.99)	495395.06 (434245.26–564297.86)	492.97 (126.64–1923.53)	982.71 (258.89–3557.08)	82.98 (78.1–87.53)	89.67 (85.4–93.77)	99.35 (77.15–114.55)
Male	51546.18 (44558.43–58362.72)	92764.61 (80484.2–104769.8)	135027.86 (117406.28–154195.77)	251258.61 (219983.71–285753.63)	231.27 (55.8–1053.94)	446.96 (109.69–1861.05)	79.96 (75.21–84.14)	86.08 (82.34–89.91)	93.26 (75.27–110.03)
Female	47929.33 (41351.85–54258.09)	89257.97 (77855.64–100334.98)	126157.48 (108828.39–144981.91)	244136.46 (213324.08–279851.93)	261.7 (72.22–923.09)	535.75 (151.81–1799.63)	86.23 (80.81–91.24)	93.52 (88.67–98.12)	104.72 (79.54–120.95)

**Table 2:** Age standardized incidence, prevalence, YLD, and annual percentage changes of headache disorders and subtypes in South Asia, 1990–2021.

	1990	2021	1990	2021	1990	2021
	ASIR (95% UI)	ASIR (95% UI)	ASPR (95% UI)	ASPR (95% UI)	ASYR (95% UI)	ASYR (95% UI)
Headache disorders						
Country/Region						
Afghanistan	9740.09 (8644.38–10775.36)	9740.19 (8655.07–10811.61)	34717.45 (31640.4–37915.48)	34515.13 (31454.04–37745.16)	666.05 (160.22–1380.24)	651.2 (157.5–1341.15)
Male	9407.06 (8234.17–10539.47)	9407.14 (8250.45–10559.13)	31511.85 (28251.69–34878.6)	31512.07 (28232.11–34834.42)	496.51 (128.57–1024.6)	499.17 (126.39–1022.47)
Female	10052.49 (8895.75–11098.05)	10052.61 (8913.38–11102.32)	37410.33 (34207.38–40967.36)	37410.68 (34232.51–40963.22)	803.86 (186.81–1675.62)	801.73 (185.41–1654.61)
Bangladesh	10133.14 (8994.91–11266.47)	10131.93 (8984.32–11256.76)	34932.41 (31959.93–38296.6)	35035.46 (32028.42–38346.95)	569.5 (93.48–1239.6)	578.7 (94–1259.19)
Male	9988.06 (8848.36–11151.2)	9988.08 (8840.08–11153.94)	33276.49 (30232.9–36951.47)	33275.57 (30230.43–36934.07)	472.42 (74.85–1018.55)	474.15 (75.51–1020.96)
Female	10277.35 (9073.71–11447.44)	10277.19 (9075.62–11449.53)	36711.72 (33506.37–40032.18)	36711.71 (33502.59–39990.4)	674.97 (114.25–1475.55)	677.67 (112.39–1477.69)
Bhutan	10124.96 (8963.43–11269.34)	10132.45 (8997.94–11275.32)	34889.71 (31,868–38278.06)	34930.69 (31973.13–38302.64)	569.98 (90.58–1237.7)	574.93 (91.68–1247.94)
Male	9988.11 (8846.06–11178.21)	9987.99 (8846.52–11153.7)	33275.97 (30229.6–36961.93)	33276.28 (30195.05–36934.19)	474.21 (77.72–1020.47)	476.58 (74.33–1030.81)
Female	10277.11 (9060.46–11442.73)	10277.19 (9083.38–11421.52)	36710.98 (33459.21–39977.21)	36711.74 (33511.14–40045.97)	678.47 (111.13–1486.61)	681.85 (110.49–1478.81)
India	10566.79 (9382.61–11662.12)	10566.3 (9373.77–11663.77)	35585.79 (32893.28–38383.4)	35625.42 (32910.28–38378.18)	587.22 (102.69–1273.16)	592.87 (104.18–1270.46)
Male	10324.7 (9113.31–11461.33)	10328.38 (9130.39–11451.06)	33,375 (30704.97–36183.36)	33419.71 (30671.13–36159.13)	478.03 (85.84–1009.3)	483.21 (84.69–1019.72)
Female	10826.24 (9672.42–11984.42)	10822.18 (9645.96–11997.84)	37981.12 (35110.67–40932.98)	37931.53 (35075.75–40813.33)	706.12 (122.68–1,555)	707.41 (124.38–1544.7)
Maldives	9940.21 (8802.13–11060.15)	9830.33 (8701.86–10969.55)	35406.41 (32362.77–38661.37)	34816.97 (31787.05–38040.18)	630.59 (93.53–1354.57)	610.25 (93.71–1320.53)
Male	9290.04 (8165.54–10404.44)	9289.8 (8187.16–10385.15)	32147.5 (29118.43–35538.8)	32147.61 (29126.66–35472.69)	511.21 (86.24–1081.78)	513.76 (87.22–1079.38)
Female	10645.61 (9487.55–11814.31)	10645.91 (9479.71–11835.44)	38999.46 (35657.43–42573.76)	38999.72 (35628.54–42525.62)	760.13 (103.58–1651.89)	764.13 (101.88–1653.95)
Nepal	10183.79 (9026.14–11367.47)	10156.03 (9053.02–11272.46)	35784.35 (32481.63–39193.72)	35687.35 (32557.76–38900.39)	609.48 (99.77–1303.04)	619.06 (102.31–1,342)
Male	10074.9 (8921.76–11275.6)	10056.53 (8848.98–11153.71)	34120.57 (30846.45–37799.54)	34020.45 (30858.76–37552.51)	507.85 (82.61–1098.44)	510.91 (81.1–1111.06)
Female	10291.73 (9128.73–11492.44)	10264.52 (9152.51–11298.39)	37431.46 (34195.34–40873.09)	37161.12 (33923.62–40424.45)	710.64 (118.29–1517.69)	712.82 (118.77–1540.51)
Pakistan	10900.7 (9717.52–12110.28)	10900.47 (9716.03–12096.98)	36641.1 (33859.82–39626.05)	36578.12 (33768.93–39508.3)	576.33 (94.14–1260.7)	563.44 (77.79–1239.86)
Male	10873.99 (9582.36–12064.33)	10873.81 (9575.49–12047.65)	35476.05 (32540.58–38615.9)	35388.62 (32432.34–38441.16)	486.68 (76.92–1063.14)	472.89 (62.02–1034.12)
Female	10931.18 (9775.58–12104.76)	10931.04 (9756.25–12,105)	37933.33 (35011.42–40706.15)	37816.07 (34906.13–40594.75)	675.98 (112.9–1478.04)	657.6 (96.14–1441.09)
Sri Lanka	9965.25 (8826.74–11083.27)	9987.48 (8851.7–11109.69)	35563.84 (32478.2–38848.25)	35698.96 (32624.77–39018.74)	636.98 (93.92–1369.82)	641.35 (93.73–1377.04)
Male	9290.15 (8189.25–10406.95)	9289.63 (8170.4–10400.43)	32147.84 (29116.12–35538.79)	32147.9 (29139.78–35511.51)	509.69 (86.66–1068.32)	508.57 (84.75–1082.93)
Female	10645.64 (9482.2–11808.98)	10645.58 (9492.97–11817.64)	38999.7 (35615.1–42553.61)	39000.21 (35647.46–42567.63)	764.94 (102.54–1658.77)	765.72 (102.88–1678.07)
South Asia	10537.63 (9353.2–11643.8)	10542.59 (9340.67–11641.19)	35628.79 (32901.92–38523.8)	35671.97 (32958.44–38469.35)	586.63 (101.16–1271.87)	590.44 (100.97–1270.95)
Male	10321.51 (9126.38–11441.08)	10336.99 (9139.63–11458.8)	33553.13 (30828.75–36489.04)	33607.3 (30877.51–36414.08)	479.7 (84.09–1018.08)	482.71 (81.63–1027.07)
Female	10768.39 (9609.56–11873.54)	10761.38 (9585.2–11861.11)	37873.65 (34936.45–40778.38)	37801.33 (34942.25–40632.76)	702.71 (121.26–1545.2)	701.33 (120.58–1523.12)
Migraine						
Afghanistan	1259.36 (1074.4–1453.99)	1219.25 (1037.68–1408.34)	14590.27 (12506.39–16711.86)	15219.55 (12883.74–17868.2)	600.21 (108.16–1316.74)	585.63 (106.64–1275.39)
Male	927.15 (783.57–1085.14)	927.15 (783.57–1085.14)	11279.44 (9442.73–13292.84)	11279.44 (9442.73–13292.84)	439.74 (82.75–948.03)	442.11 (86.24–945.79)
Female	1524.89 (1299.5–1752.34)	1524.89 (1299.5–1752.34)	19136.53 (16242.76–22511.03)	19136.53 (16242.76–22511.03)	730.27 (127.86–1601.79)	728.07 (126.46–1590.62)
Bangladesh	1208.43 (1029.1–1385.25)	1219.24 (1039.6–1397.43)	14824.95 (12760.81–16953.82)	14622.91 (12354.85–17096.06)	521.53 (64.26–1164.16)	529.83 (65.93–1188.38)
Male	999.03 (846.15–1157.28)	999.03 (846.15–1157.28)	11797.45 (9963.83–13866.33)	11797.45 (9963.83–13866.33)	429.18 (52.15–977.54)	430.37 (52.17–985.77)
Female	1431.33 (1,228–1650.46)	1431.33 (1,228–1650.46)	17295.49 (14626.42–20341.37)	17295.49 (14626.42–20341.37)	621.73 (77.51–1372.06)	623.93 (78.91–1380.71)
Bhutan	1201.85 (1023.53–1377.96)	1208.09 (1029.06–1385.08)	16084.72 (13857.54–18345.74)	14430.27 (12190.24–16863.35)	521.78 (64.55–1168.67)	526.35 (64.36–1188.32)
Male	999.03 (846.15–1157.28)	999.03 (846.15–1157.28)	11797.45 (9963.83–13866.33)	11797.45 (9963.83–13866.33)	430.79 (51.55–979.27)	432.77 (51.5–987.44)
Female	1431.33 (1,228–1650.46)	1431.33 (1,228–1650.46)	17295.49 (14626.42–20341.37)	17295.49 (14626.42–20341.37)	624.96 (76.51–1398.89)	628.08 (77.53–1412.84)
India	1231.68 (1085.77–1382.46)	1232.92 (1080.89–1385.31)	14374.3 (12145.85–16792.63)	14909.22 (12770.95–16967.7)	536.03 (69.56–1181.73)	540.99 (70.28–1209.87)
Male	991.52 (861.72–1119.92)	994.32 (867.02–1127.06)	11822.27 (10196.84–13591.92)	11882.27 (10,134–13674.14)	431.97 (58.42–955.42)	436.64 (57.83–972.74)
Female	1492.82 (1321.46–1679.28)	1487.79 (1307.14–1670.63)	18146.8 (15603.83–20728.2)	18072.32 (15623.47–20718.35)	649.34 (82.59–1454.35)	650.08 (83–1460.8)
Maldives	1281.38 (1090.13–1481.61)	1238.55 (1054.1–1436.81)	15558.3 (13173.32–18335.58)	14991.69 (12756.93–17610.83)	582.29 (64.57–1318.97)	562.08 (63.1–1277.98)
Male	1022.29 (859.06–1188.2)	1022.29 (859.06–1188.2)	12381.06 (10507.22–14584.49)	12381.06 (10507.22–14584.49)	466.55 (58.07–1022.61)	468.69 (57.93–1041.18)
Female	1551.4 (1326.03–1792.8)	1551.4 (1326.03–1792.8)	19173.28 (16180.69–22503.82)	19173.28 (16180.69–22503.82)	707.68 (68.77–1627.37)	711.22 (70.71–1628.95)
Nepal	1264.39 (1073.76–1460.98)	1272.83 (1086.72–1,469)	15768.45 (13398.97–18578.29)	15717.9 (13261.38–18538.76)	559.27 (68.67–1240.87)	568.37 (70.18–1262.36)
Male	1050.77 (894.82–1215.18)	1049.03 (888.98–1222.21)	12825.13 (10597.56–15215.59)	12802.91 (10758.72–15166.78)	462.84 (55.6–1053.36)	465.66 (55.97–1056.89)
Female	1478.01 (1257.63–1690.35)	1476.36 (1255.65–1717.77)	18273.32 (15671.28–21331.3)	18239.49 (15418.62–21459.75)	655.21 (83.1–1438.92)	657.45 (80.27–1464.71)
Pakistan	1218.04 (1072.69–1360.86)	1223.13 (1077.41–1366.83)	14850.77 (12801.29–16953.83)	14570.79 (12494.14–16671.84)	526.45 (64.99–1181.42)	517.34 (52.91–1172.44)
Male	1023.54 (898.4–1141.75)	1023.54 (898.4–1141.75)	12176.62 (10401.91–14178.21)	12087.13 (10319.39–14082.73)	441.54 (51.77–982.23)	431.16 (41.06–969.11)
Female	1431.15 (1260.84–1605.74)	1431.15 (1260.84–1605.74)	17269.26 (14826.02–19,640)	17148.78 (14715.8–19511.1)	620.77 (78.71–1397.01)	606.93 (65.97–1375.91)
Sri Lanka	1285.6 (1094.36–1485.74)	1290.9 (1099.14–1,491)	15638.53 (13284.73–18430.24)	15890.54 (13502.71–18725.41)	588.28 (65.43–1340.67)	592.23 (65.81–1349.2)
Male	1022.29 (859.06–1188.2)	1022.29 (859.06–1188.2)	12381.06 (10507.22–14584.49)	12381.06 (10507.22–14584.49)	464.99 (59.83–1027.45)	463.78 (60.66–1020.85)
Female	1551.4 (1326.03–1792.8)	1551.4 (1326.03–1792.8)	19173.28 (16180.69–22503.82)	19173.28 (16180.69–22503.82)	712.22 (70.64–1635.56)	712.63 (70.88–1658.11)
South Asia	1229.93 (1083.77–1385.47)	1232.12 (1080.34–1386.43)	15624.3 (13231.58–18367.97)	14880.83 (12,725–16995.05)	535.76 (69–1181.79)	539.3 (68.45–1201.52)
Male	997.04 (865.69–1127.35)	999.69 (870.72–1133.87)	11879.49 (10224.32–13699.57)	11919.54 (10174.28–13722.89)	433.92 (57.7–958.88)	436.78 (56.27–968.63)
Female	1481.67 (1307.95–1664.5)	1476.39 (1294.62–1656.27)	18020.85 (15455.02–20678.67)	17929.66 (15411.52–20506.6)	646.3 (82.07–1439.42)	644.91 (80.81–1438.59)
TTH						
Afghanistan	8480.73 (7407.25–9525.1)	8520.94 (7419.07–9605.02)	23482.77 (20268.79–27275.59)	23578.38 (20370.41–27419.47)	65.85 (21.53–189.8)	65.57 (21.59–192.9)
Male	8479.92 (7326.64–9590.26)	8479.99 (7336.38–9615.55)	23378.88 (20008.12–27314.1)	23379.17 (20062.28–27289.19)	56.78 (16.86–178.93)	57.06 (16.58–177.55)
Female	8527.61 (7437.02–9635.48)	8527.72 (7455.11–9620.57)	23670.95 (20449.35–27410.79)	23671.69 (20414.96–27455.02)	73.59 (25.28–199.6)	73.66 (25.26–197.77)
Bangladesh	8924.71 (7778.47–10052.47)	8912.69 (7778.73–10033.24)	24809.12 (21195.91–28767.96)	24771.44 (21,160–28698.1)	47.97 (12.27–180.03)	48.87 (12.84–175.36)
Male	8989.03 (7849.59–10126.55)	8989.05 (7842.64–10132.61)	25062.98 (21308.17–29200.37)	25061.89 (21342.44–29173.47)	43.24 (10.33–191.51)	43.78 (10.3–183.04)
Female	8846.02 (7676.08–10016.7)	8845.86 (7685.31–10021.38)	24504.98 (20896.77–28375.11)	24505.03 (20955.38–28388.52)	53.24 (14.62–176)	53.74 (14.91–177.82)
Bhutan	8923.11 (7792.69–10036.22)	8924.36 (7795.91–10044.27)	24802.78 (21159.45–28816.4)	24804.7 (21191.89–28828.32)	48.2 (12.52–185.34)	48.58 (12.9–178.81)
Male	8989.08 (7850.31–10148.19)	8988.96 (7839.33–10151.54)	25062.42 (21284.94–29259.81)	25062.76 (21308.44–29177.02)	43.43 (10.07–195.88)	43.81 (10.29–186.59)
Female	8845.78 (7661.79–10025.43)	8845.86 (7688.57–10018.79)	24504.04 (20889.85–28338.59)	24504.97 (20944.17–28367.8)	53.51 (15.46–177.14)	53.77 (15.47–179.51)
India	9335.11 (8162.27–10430.63)	9333.37 (8160.49–10426.38)	25300.04 (22420.05–28440.68)	25298.04 (22421.85–28418.03)	51.19 (14–187.37)	51.88 (14.09–180.56)
Male	9333.18 (8124.16–10497.85)	9334.06 (8122.76–10491.88)	25190.6 (22216.47–28268.31)	25194.71 (22230.99–28253.08)	46.05 (11.97–194.57)	46.57 (11.72–187.63)
Female	9333.42 (8169.03–10443.02)	9334.4 (8170.01–10444.33)	25405.48 (22323.84–28719.54)	25409.13 (22317.76–28701.96)	56.78 (16.64–186.57)	57.33 (16.52–186.45)
Maldives	8658.83 (7528.74–9766.76)	8591.78 (7477.04–9695.04)	24480.33 (20993.54–28121.49)	24278.21 (20796.04–27850.09)	48.3 (12.8–183.43)	48.16 (12.91–175.8)
Male	8267.75 (7159.17–9373.91)	8267.52 (7169.84–9352.65)	23261.16 (19716.38–26884.11)	23261.35 (19740.69–26879.15)	44.66 (11.6–190.34)	45.07 (11.84–175.57)
Female	9094.21 (7937.87–10246.29)	9094.51 (7941.09–10255.04)	25859.22 (22188.51–29600.45)	25859.82 (22176.16–29594.95)	52.45 (14.2–190.41)	52.91 (14.44–190.27)
Nepal	8919.39 (7758.23–10099.96)	8883.19 (7746.28–9966.79)	24882.05 (21370.15–28866.56)	24613.27 (20956.66–28522.19)	50.21 (13.87–190.46)	50.69 (13.73–179.81)
Male	9024.13 (7877.25–10233.3)	9007.5 (7806.69–10117.62)	25210.42 (21611.13–29411.51)	25109.01 (21431.67–29075.14)	45.01 (10.74–200.45)	45.25 (10.6–186.56)
Female	8813.71 (7618.83–9972.11)	8788.16 (7681.5–9850.47)	24543.28 (21136.85–28234.25)	24215.5 (20688.46–28195.99)	55.44 (16.62–181.3)	55.37 (16.45–177.17)
Pakistan	9682.66 (8507.1–10830.69)	9677.34 (8501.33–10819.06)	26767.91 (23733.68–30269.31)	26707.03 (23686.83–30189.12)	49.87 (12.81–192.76)	46.1 (11.06–181.63)
Male	9850.44 (8551.97–11013.8)	9850.27 (8545.09–11002.59)	27356.1 (24301.65–30954.51)	27322.78 (24291.17–30917.63)	45.14 (10.15–203.36)	41.73 (8.68–195.48)
Female	9500.03 (8389.78–10662.38)	9499.89 (8373.73–10689.03)	26113.2 (22972.97–29450.05)	26068.49 (22940.39–29415.51)	55.22 (15.47–188.57)	50.68 (13.5–187.27)
Sri Lanka	8679.65 (7551.48–9766.59)	8696.58 (7579.93–9778.11)	24556.79 (21053.11–28174.26)	24617.15 (21076.29–28209.67)	48.7 (13.2–178.54)	49.12 (12.83–178.26)
Male	8267.86 (7180.94–9342.36)	8267.34 (7160.53–9361.16)	23261.56 (19765.38–26853.76)	23261.64 (19730.8–26872.76)	44.7 (11.44–178.23)	44.79 (11.1–170.84)
Female	9094.24 (7935.99–10271.71)	9094.18 (7947.95–10253.36)	25859.71 (22157.43–29546.92)	25860.36 (22086.76–29625.32)	52.73 (14.29–186.07)	53.09 (14.44–188.21)
South Asia	9307.7 (8123.5–10392.62)	9310.48 (8122.13–10390.72)	25364.31 (22360.24–28562.22)	25367.73 (22378.64–28549.66)	50.86 (13.87–186.71)	51.13 (13.68–180.42)
Male	9324.47 (8103.25–10459.44)	9337.3 (8112.46–10464.22)	25345.64 (22359.2–28503.33)	25376.8 (22405.16–28540.82)	45.78 (11.83–194.57)	45.93 (11.55–186.92)
Female	9286.72 (8118.63–10374.85)	9284.99 (8116.21–10364.88)	25374.18 (22236.66–28655.72)	25362.76 (22215.54–28602.68)	56.41 (16.47–185.79)	56.42 (16.16–185.96)

### 3.2 Joinpoint regression analysis of headache disorders in South Asia

#### 3.2.1 Incidence

**Figure 2:**
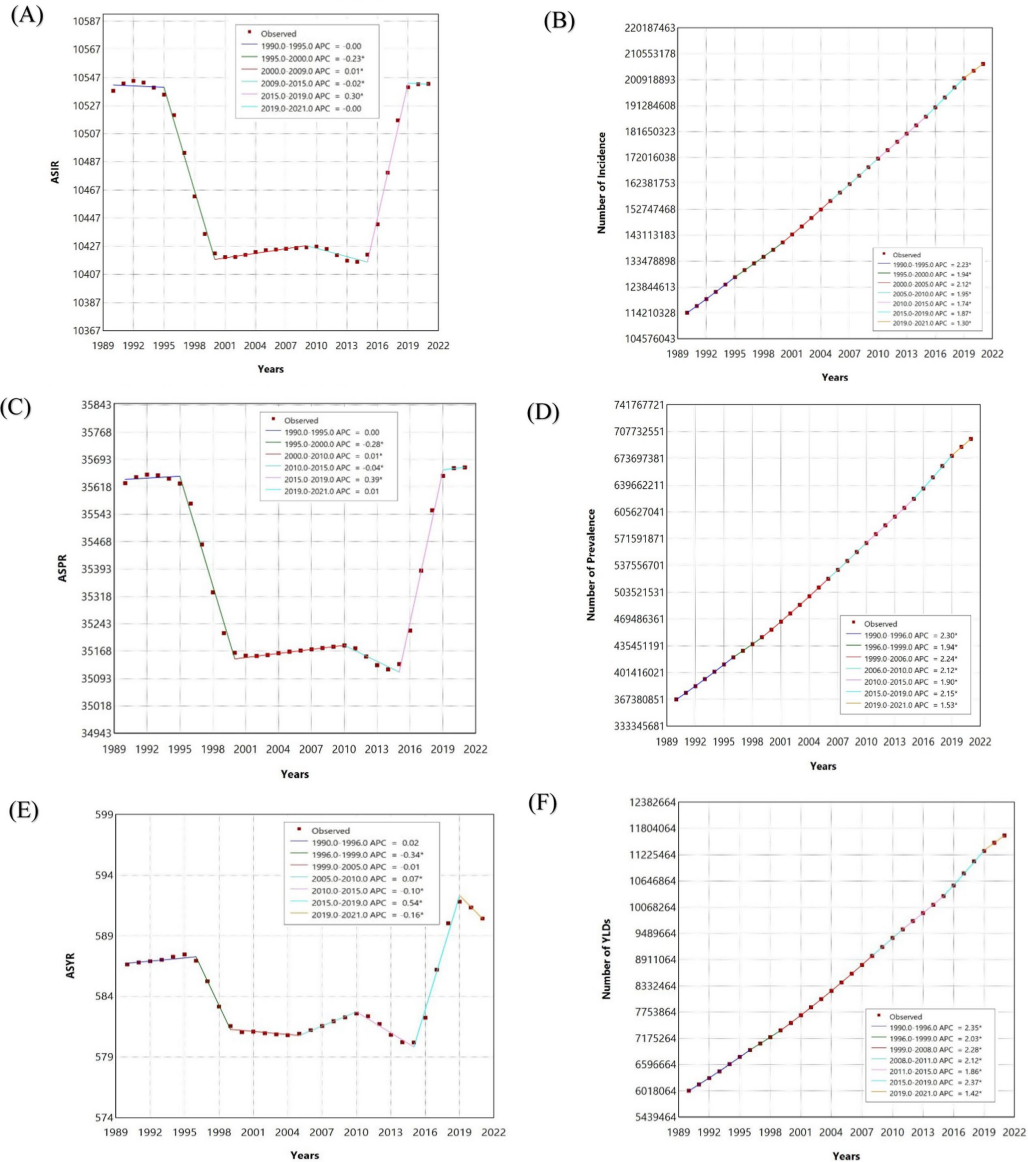
The joinpoint regression analysis of South Asia incidence, prevalence and years lived with disability (YLDs) of Headache disorders from 1990 to 2021. **(A)** The joinpoint regression analysis on the Age-Standardized Rates of incidence; **(B)** The joinpoint regression analysis on the number of incidence; **(C)** The joinpoint regression analysis on the Age-Standardized Rates of prevalence; **(D)** The joinpoint regression analysis on the number of prevalence; **(E)** The joinpoint regression analysis on the Age-Standardized Rates of YLDs; **(F)** The joinpoint regression analysis on the number of YLDs.

ASIR remained stable, fluctuating between 10,420 and 10,540 per 100,000 population. Trends included a slight decline from 1995 to 2000 (APC = −0.23%), a marginal increase from 2000 to 2009 (APC = 0.01%), and a significant rise from 2015 to 2019 (APC = 0.30%), with stabilization from 2019 to 2021 (APC = 0.00%) (Figures 2A,B). However, the absolute number of new cases rose consistently from 114.2 million in 1990 to 206.8 million in 2021, reflecting an 80.9% cumulative growth. The highest growth rate occurred between 1990 and 1995 (APC = 2.23%), with a gradual slowdown to 1.30% from 2019 to 2021 (Table 3).

**Table 3:** Age and gender specific incidence rates (per 100,000) and male-to-female ratios for headache disorders across south Asian countries in 2021.

Country	Male (95% UI)	Female (95% UI)	Male–female ratio
Afghanistan	8479.99 (7336.38–9615.55)	8527.72 (7455.11–9620.57)	0.99
Bangladesh	8989.05 (7842.64–10132.61)	8845.86 (7685.31–10021.38)	1.02
Bhutan	8988.96 (7839.33–10151.54)	8845.86 (7688.57–10018.79)	1.02
India	9334.06 (8122.76–10491.88)	9334.4 (8170.01–10444.33)	1.00
Maldives	8267.52 (7169.84–9352.65)	9094.51 (7941.09–10255.04)	0.91
Nepal	9007.5 (7806.69–10117.62)	8788.16 (7681.5–9850.47)	1.02
Pakistan	9850.27 (8545.09–11002.59)	9499.89 (8373.73–10689.03)	1.04
Sri Lanka	8267.34 (7160.53–9361.16)	9094.18 (7947.95–10253.36)	0.91
South Asia	9337.3 (8112.46–10464.22)	9284.99 (8116.21–10364.88)	1.01

#### 3.2.2 Prevalence

The prevalence analysis revealed stability in the ASPR, which fluctuated within 35,128 to 35,672 per 100,000 population. From 1990 to 1995, there was no significant change (APC = 0.00%), followed by a decline from 1995 to 2000 (APC = −0.28%) (Figures 2C,D). A minimal increase was noted from 2000 to 2010 (APC = 0.01%), followed by a small decline from 2010 to 2015 (APC = −0.04%). A significant increase was recorded from 2015 to 2019 (APC = 0.39%), with stabilization from 2019 to 2021 (APC = 0.01%). Meanwhile, the absolute number of prevalence cases surged from 367.4 million in 1990 to 698.5 million in 2021, showing a 90.1% cumulative growth. The highest growth occurred between 1990 and 1996 (APC = 2.30%), with consistent growth across subsequent intervals, ranging from 1.53 to 2.24%.

#### 3.2.3 Years lived with disability (YLDs)

The analysis of YLDs due to headache disorders revealed minor fluctuations in the ASYR, which remained relatively stable between 579.0 and 591.8 per 100,000 population. From 1990 to 1996, there was no significant change (APC = 0.02%), followed by a decline from 1996 to 1999 (APC = −0.34%) and stabilization from 1999 to 2005 (APC = −0.01%) (Figures 2E,F). A minor increase was observed from 2005 to 2010 (APC = 0.07%), followed by a decline from 2010 to 2015 (APC = −0.10%). A notable rise was recorded between 2015 and 2019 (APC = 0.54%), with a slight decline from 2019 to 2021 (APC = −0.16%). In absolute terms, the number of YLD cases rose from 6.0 million in 1990 to 11.6 million in 2021, reflecting a 93.4% growth. Growth rates were highest between 1990 and 1996 (APC = 2.35%) and remained significant across subsequent intervals, ranging from 1.42 to 2.37%.

### 3.3 Trends in age-standardized rates of migraine and tension-type headache

**Figure 3:**
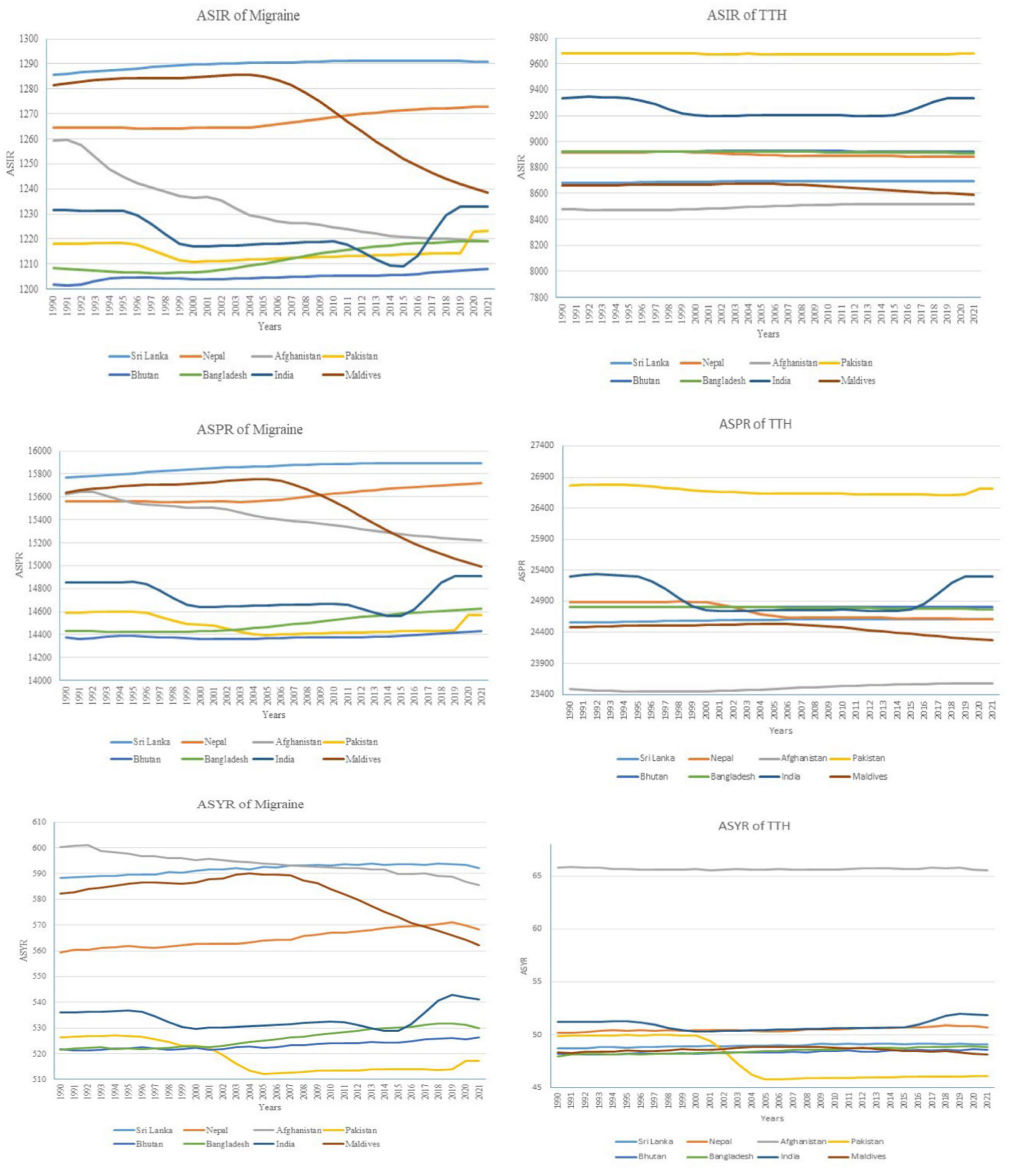
Trends in age-standardized rates of migraine and tension-type headache across south Asian countries from 1990 to 2021.

The ASIR of migraine across South Asia showed predominantly stable trends from 1990 to 2021. Sri Lanka increased slightly from 1285.60 to 1290.90, Nepal from 1264.39 to 1272.83, and Bhutan from 1201.85 to 1208.09 (Figure 3). Afghanistan experienced a decline from 1259.36 to 1219.25, while the Maldives showed the most notable decrease from 1281.38 to 1238.55. Pakistan’s ASIR fluctuated, ending slightly higher at 1223.13, and India saw minimal change, rising from 1231.68 to 1232.92. Bangladesh showed a minor increase from 1208.43 to 1219.24. ASPR of migraine also displayed stable trends. Sri Lanka rose from 15768.45 to 15890.54, Nepal from 15558.30 to 15717.90, and India from 14850.77 to 14909.22. Afghanistan experienced a notable decline from 15624.30 to 15219.55, while Pakistan fluctuated slightly, ending at 14570.79. Bhutan, Bangladesh, and the Maldives showed minor fluctuations without significant change. The ASYR for migraine showed mixed trends. Sri Lanka’s ASYR slightly decreased from 588.28 to 592.23, and Nepal varied minimally from 559.27 to 568.37. Afghanistan, Pakistan, Bhutan, Bangladesh, and the Maldives showed minor declines, with Afghanistan decreasing from 600.21 to 585.63. India was the exception, with a slight increase from 536.03 to 540.99. For TTH, Pakistan consistently reported the highest ASIR, peaking at 9677.81 in 2020 and marginally decreasing to 9677.34 by 2021. Afghanistan’s ASIR increased steadily from 8480.73 to 8520.94. Bhutan, Nepal, and Sri Lanka maintained stable ASIRs around the 8,900 range. ASPR trends for TTH followed a similar pattern, with Pakistan showing the highest values and slight fluctuations, while Sri Lanka, Nepal, and Afghanistan reported lower but stable rates.

## 4 Age and gender-specific analysis in South Asia

**Figure 4:**
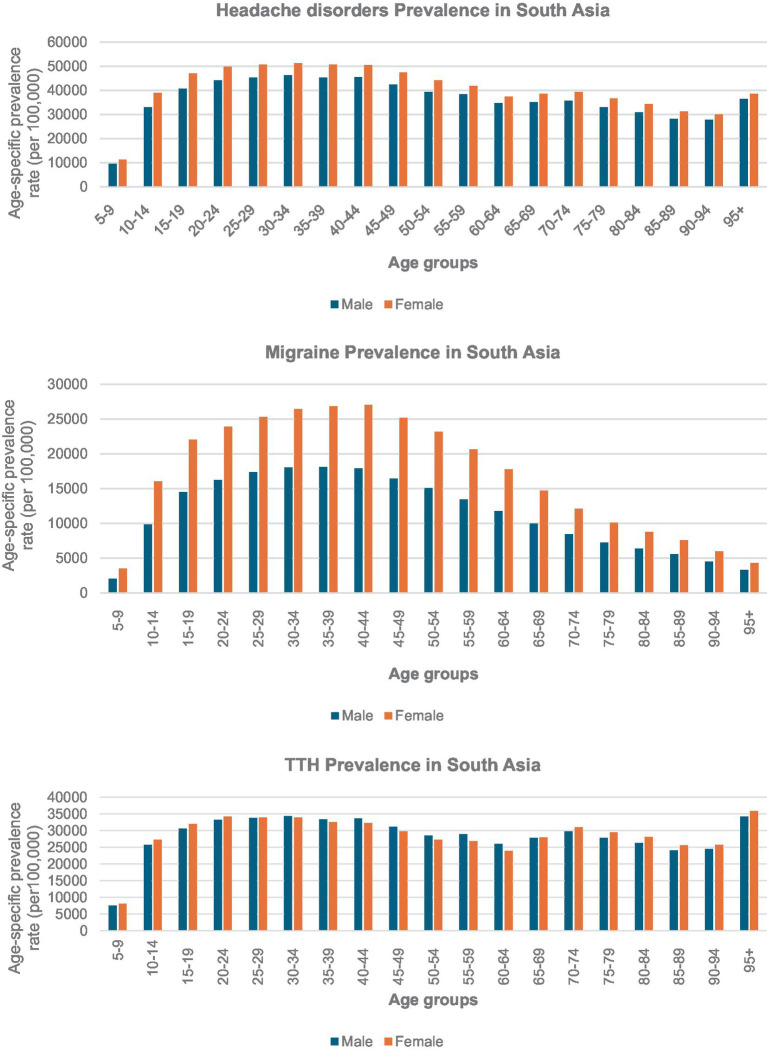
Age-specific prevalence of headache disorders, migraine and tension-type headache (TTH) in south Asia by gender, 2021.

The prevalence analysis of headache disorders in South Asia, including their subtypes migraine and TTH, reveals distinct patterns across age and gender groups. Headache disorders prevalence among females aged 30–34 years is 51,332.96 per 100,000 and males in the same age group is 46,203.64 per 100,000 (Figure 4). Prevalence across middle-aged groups begin decline significantly after the age of 55, with the rates observed in the 90–94 age group is 27,839.75 per 100,000 for males and 30,077.76 per 100,000 for females. For the migraine subtype, the prevalence in females aged 40–44 years (27,016.05 per 100,000) and in males aged 35–39 years (18,113.81 per 100,000). After these peak age ranges, the prevalence steadily decreases, reaching 3,312.58 per 100,000 for males and 4,320.85 per 100,000 for females in the 95+ age group. In contrast, TTH, prevalence is observed among males aged 30–34 years (34,368.01 per 100,000) and females aged 25–29 years (33,841.92 per 100,000). A unique resurgence is seen in the oldest age group (95+ years), with TTH prevalence reaching 34,173.33 per 100,000 for males and 35,825.53 per 100,000 for females.

The analysis of headache disorder data from 1990 to 2021 in South Asia reveals distinct trends in the incidence, prevalence, and disability impact between male and female populations. For headache disorders, the male incidence increased from 57,715.26 to 103,067.95 per 100,000, an increase of 78.58%, while female incidence rose from 56,519.97 to 103,737.67 per 100,000, marking an 83.54% increase (Table 1). Similarly, the YLD for males increased from 2,546.95 to 4,831.76 per 100,000, and for females from 3,475.59 to 6,816.32 per 100,000 over the same period.For migraine disorders specifically, the male population saw an incidence rise from 6,169.08 to 10,303.35 per 100,000 (67.02%), and females from 8,590.65 to 14,479.7 per 100,000 (68.55%). YLD for males in this category increased from 2,315.68 to 4,384.8 per 100,000, while for females it escalated from 3,213.89 to 6,280.57 per 100,000. For TTH, the incidence among males increased from 51,546.18 to 92,764.61 per 100,000, a growth of 79.96%, and in females from 47,929.33 to 89,257.97 per 100,000, an increase of 86.23%. YLD for males rose from 231.27 to 446.96 per 100,000, and for females from 261.7 to 535.75 per 100,000.

The study of age-standardized incidence rates (ASIR), prevalence (ASPR), and years lived with disability (YLD) for headache disorders in South Asia from 1990 to 2021 showed minimal changes over the three decades. In Afghanistan, the male ASIR started at 9,407.06 per 100,000 in 1990 and slightly rose to 9,407.14 per 100,000 by 2021 (Table 2). Females began with an ASIR of 10,052.49 per 100,000 in 1990, slightly changing to 10,052.61 per 100,000 in 2021. In Bangladesh, the female ASPR remained steady at 36,711.71 per 100,000 from 1990 to 2021, the male ASPR slightly decreased from 33,276.49 to 33,275.57 per 100,000 in the same period. YLD for males in Nepal showed a slight increase from 507.85 per 100,000 in 1990 to 510.91 per 100,000 in 2021, indicating a mild increase in disability attributed to headaches.

## 5 Discussion

The burden of headache disorders, including migraines and TTH, has increased substantially in South Asia from 1990 to 2021. Absolute incidence rose by 81.03%, prevalence by 90.1%, and YLDs by 93.41%, while age-standardized rates (ASIR, ASPR, and ASYR) remained relatively stable across the study period, demonstrating minimal regional fluctuations. In absolute terms, new cases of headache disorders grew from 114.2 million in 1990 to 206.8 million in 2021, while prevalence surged from 367.4 million to 698.5 million, demonstrating a dramatic rise in the burden. Global studies corroborate observed trends in headache disorders by documenting a significant global rise in age-standardized incidence, prevalence, and YLDs with contributions from South Asia significantly impact these global figures (16–18). Headache disorders represent a significant and increasing health concern in South Asia. The region experienced not only substantial growth in absolute cases but also notable disparities in burden among countries. Afghanistan showed the highest relative increases in all metrics, with prevalence and YLDs more than doubling. Meanwhile, India, the most populous country in the region, contributed the largest absolute burden, with its prevalence increasing by over 240 million cases from 1990 to 2021. This trend highlights the dual impact of population growth and differential healthcare access across the region (19–21). The increase in the absolute numbers of headache disorders in South Asia, despite stable age-standardized rates over the years, may be linked to several potential precipitants and aggravating factors, including stress, inadequate sleep, irregular, poor dietary habits (34, 35). These elements have been frequently associated with the onset or intensification of headache symptoms, particularly in individuals with migraine and tension-type headaches (TTH). Additionally, alcohol consumption and hormonal fluctuations, such as those occurring during menstruation, have been reported as possible triggers for headache episodes in certain individuals (36). However, the precise mechanisms through which these elements influence headache development and progression remain complex and require further investigation. Despite stable age-standardized rates, the sharp increase in absolute cases of migraines underscores the persistent challenge they pose, especially in regions with underdeveloped healthcare systems. Additionally, enhanced awareness and progress in diagnostic techniques may have improved reporting, further inflating these numbers (16). However, persistent disparities in healthcare access, especially in low-resource areas like Afghanistan, significantly worsen the overall burden of these disorders exacerbate the challenges associated with managing headache disorders. Limited access to diagnostic and treatment facilities significantly increases the burden of these conditions. Moreover, the unavailability of advanced treatments and medications exacerbates the challenges, leading to heightened disability levels and economic hardships (10). From 1990 to 2021, our study identified a persistent and significant growth in the burden of migraine headaches across South Asia, with a marked 91.85% increase in prevalence and a 92.88% rise in YLDs, indicating a severe and escalating impact on public health in the region. Similar to our findings, a study reported a significant increase in the prevalence of migraines in South Asia, highlighting a substantial rise from previous years (2). Another research by Wang et al. (22) indicated a high age-standardized YLD rate for migraines in economically disadvantaged regions, demonstrating a severe impact on daily life and work. These observations from other studies corroborate our results, emphasizing the widespread and escalating burden of migraines across various regions. Its burden varied significantly across countries in South Asia. Afghanistan experienced the largest relative increases in all metrics, reflecting severe socio-economic and healthcare challenges. By contrast, the Maldives showed reductions in age-standardized rates for both migraines and TTH, suggesting potential improvements in healthcare delivery or demographic shifts. India bore the highest absolute burden, contributing the majority of cases and YLDs, highlighting the scale of the challenge in the region’s largest country which is contributing significantly for this rise. Pakistan reported consistently high age-standardized rates for TTH, further emphasizing its unique burden of this subtype. Meanwhile, Bangladesh demonstrated significant increases in YLDs, underscoring the growing disability impact of headache disorders in this country. In Bangladesh, the rising prevalence of conditions such as migraines may be influenced by factors like rapid urbanization and associated lifestyle changes (23). These disparities highlight the need for tailored strategies to address the varying challenges across the region. TTH exhibited even greater growth than migraines, with absolute incidence increasing by 82.98%, prevalence by 89.67%, and YLDs by 99.35%. Other studies indicate that this increase can be attributed to multiple factors, including the high prevalence of TTH, its persistent nature, and the challenges related to its identification and treatment (24, 25). Pakistan consistently reported the highest age-standardized rates for TTH, while Afghanistan saw steady increases in incidence and prevalence. The Maldives experienced the steepest relative increase in TTH-related YLDs, with a dramatic rise of 242.47%, highlighting the growing impact of TTH on disability metrics in smaller countries. These findings highlight the need for targeted interventions addressing both the overall burden of headache disorders and the specific patterns observed in their subtypes across these regions.

Headache disorders and its subtypes demonstrated marked disparities across age and gender, with females consistently reporting higher prevalence rates than males, particularly in middle-aged women. Similar to the other global findings which shows the greatest ASPR in the 40–44 age group of females, we also found for migraines, prevalence peaked in females aged 40–44 years. The highest burden was observed in this age group in South Asia, which is linked to hormonal changes and differences in healthcare access or health-seeking behavior (26–28). Unlike migraines, TTH affected females most significantly in the 25–29 age group. Rastogi et al. (29) study suggests that TTH typically manifest at a younger age in females compared to males, indicating a gender-specific predisposition for earlier onset of TTH. TTH prevalence displayed a unique pattern, peaking in earlier working-age groups and showing a resurgence among the elderly, particularly those aged 95 and above. This pattern suggests that TTH might have different risk factors compared to migraines, potentially linked to long-term occupational strain or reduced healthcare access in older populations. The widespread and sustained prevalence of TTH underscores its significant contribution to the overall burden of headache disorders. Other studies suggest that this disparity is observed in both the frequency and intensity of these conditions, particularly in migraines, where biological factors such as hormonal fluctuations and the trigeminovascular system’s activation play a crucial role. Migraines are three to four times more common in females than in males, with women reporting more severe symptoms and greater disability due to these headaches (30–33).

In the case of TTH, while females generally show higher prevalence, the gender differences are less significant compared to migraines. Nonetheless, lifestyle factors such as physical exercise, stress, and socioeconomic background, screen time affect the intensity of TTH in women more profoundly. The evidence highlights the importance of developing gender-sensitive health interventions, as women are more vulnerable to the impact of headache disorders due to a combination of biological, lifestyle, and socioeconomic factors (37). Tailored healthcare strategies are essential to address these disparities and improve treatment outcomes for women. The study’s strengths include its use of comprehensive, long-term data from the GBD study, offering valuable insights into headache and its subtypes trends over three decades. The regional focus allows for the identification of country-specific variations, while the use of standardized metrics such as incidence, prevalence, and YLD ensures consistency in data comparison. Additionally, the analysis of gender disparities sheds light on the disproportionate impact of migraines on women. However, the study has limitations, including the limited headache-focused healthcare in South Asian countries, which may contribute to underdiagnosis and underreporting of migraine and tension-type headaches, potentially affecting the accuracy of the data. It relies on modeled estimates, which may introduce inaccuracies due to data gaps and limited healthcare access. Additionally, cultural differences in healthcare-seeking behavior, particularly regarding stigma around headaches, are not accounted for, which may skew prevalence estimates. Future research should also explore the role of socioeconomic factors in shaping the epidemiology of headache disorders in this diverse region.

## 6 Conclusion

The study highlights the substantial and increasing burden of headache disorders, including migraines and TTH, across South Asia from 1990 to 2021, underscoring their critical public health implications. The findings reveal significant growth in absolute incidence, prevalence, YLDs, with pronounced variation between countries. Afghanistan recorded the highest relative increases, reflecting severe healthcare challenges, while India bore the highest absolute burden for both migraines and TTH, emphasizing its pivotal role in the regional burden. Migraines emerged as a major contributor to the overall disability burden, with notable increases in countries such as Afghanistan and India, whereas TTH demonstrated faster growth in absolute metrics, reflecting its persistent nature and widespread prevalence. The analysis further identified key demographic patterns, with women and middle-aged adults disproportionately affected, emphasizing the need for targeted interventions. These findings underscore the necessity for region-specific, scalable public health strategies tailored to the diverse challenges of individual countries and subtypes, ensuring an effective response to mitigate the disabling impact of headache disorders across South Asia.

## Data Availability

The data that support the findings of this study are openly available in Global Burden of Disease Study 2021 (GBD 2021) at https://vizhub.healthdata.org/gbd-results/.

## Footnotes

^1^ http://ghdx.healthdata.org/gbd-results-tool
